# Epstein–Barr Virus-Positive T/NK-Cell Lymphoproliferative Disorders Manifested as Gastrointestinal Perforations and Skin Lesions: A Case Report

**DOI:** 10.1097/MD.0000000000002676

**Published:** 2016-02-08

**Authors:** Hai-Juan Xiao, Ji Li, Hong-Mei Song, Zheng-Hong Li, Mei Dong, Xiao-Ge Zhou

**Affiliations:** From the Department of Pediatrics, Peking Union Medical College Hospital, Peking Union Medical College, Chinese Academy of Medical Sciences, Beijing, China (X-HJ, L-J, S-HM, L-ZH, D-M); and Department of pathology, Beijing Friendship Hospital, Capital Medical University, Beijing, China (Z-XG).

## Abstract

Systemic Epstein–Barr virus (EBV)-positive T-cell lymphoproliferative disorders (LPDs) of childhood is a highly aggressive EBV-positive T/natural killer (NK)-cell LPD, which emerges in the background of chronic active EBV infection (CAEBV) or shortly after primary acute EBV infection. The clinical presentations of CAEBV are varied; patients with atypical manifestations are easily misdiagnosed. We described a 14-year-old boy suffering from digestive disorders and intermittent fever for 1 year and 9 months, whose conditions worsened and skin lesions occurred 2 months before hospitalization. He was diagnosed as inflammatory bowel diseases (IBD) and treated accordingly. His other clinical features, hepatosplenomegaly, lymphadenopathy, anemia, hypoalbuminemia, and elevated inflammatory marks, were found in hospitalization. The boy suffered from repeatedly spontaneous intestinal perforations shortly after hospitalization and died of intestinal hemorrhea. The pathological results of intestine and skin both showed EBV-positive T/NK-cell LPD (lymphoma stage).

There are rare studies reporting gastrointestinal perforations in EBV-positive T/NK-cell LPD, let alone repeatedly spontaneous perforations. Based on the clinical features and pathological results of this patient, the disease progressed from CAEBV (T-cell type) to systemic EBV-positive T-cell LPD of childhood (lymphoma). Not all the patients with CAEBV could have unusual patterns of anti-EBV antibodies. However, the presence of high EBV loads (EBV-encoded early small ribonucleic acid (RNA) (EBER) in affected tissues and/or EBV deoxyribonucleic acid (DNA) in peripheral blood) is essential for diagnosing CAEBV. Maybe because of his less common clinical features for CAEBV and negative anti-EBV antibodies, the boy was not diagnosed correctly. We should have emphasized the test for EBER or EBV-DNA. Meanwhile, for the IBD patients whose manifestations were not typical, and whose conditions were not improved by suitable therapies against IBD, infectious and malignant diseases should be considered.

## INTRODUCTION

Epstein–Barr virus (EBV), also called human herpes virus 4 (HHV-4), is a liner, double-stranded deoxyribonucleic acid (DNA) virus. It is estimated that over 90% of humans are infected by EBV and the infection persists for life.^1^ Primary infections are usually asymptomatic in childhood, but a self-limiting infectious mononucleosis (IM) syndrome happens to approximately one-third of the cases in adolescence or adulthood.^2^ In some hosts, chronic active EBV infection (CAEBV) characterized by persistent or recurrent IM-like symptoms (fevers, hepatosplenomegaly, and lymphadenopathy) might develop.^3^ Infection with EBV also has been implicated in the development of a variety of malignancies including EBV associated B-cell lymphomas and EBV associated T/natural killer (NK)-cell lymphomas.^1^

EBV-positive T/NK-cell lymphoproliferative disorders (LPDs) encompasses a heterogeneous group of disorders that share the feature of clonal expansion of EBV-infected T or NK cells: systemic EBV-positive T-cell LPD of childhood, hydroa vacciniforme-like lymphoma, CAEBV, EBV-hemophagocytic lymphohistiocytosis (HLH), hydroa vacciniforme (HV), and hypersensitivity to mosquito bites (HMB).^4^ Among these, the front 2 kinds occur almost entirely in children, and are included in the 2008 World Health Organization (WHO) classification of lymphomas.^5^ Systemic EBV-positive T-cell LPD of childhood is a highly aggressive condition with rapid evolution to multiple-organ failure and death, which emerges in the background of CAEBV or shortly after primary acute EBV infection.^5^

It is known that several primary and secondary immunodeficiencies are representatively vulnerable to EBV infections.^6^ However, there is no apparent immunodeficiency or certain genetic predisposition for CAEBV individuals, though CAEBV appears to be more prevalent in East Asian and Mexican ethnicity.^6^ The clinical presentations of CAEBV are varied; major symptoms are fever, lymphadenopathy, hepatomegaly, splenomegaly, liver dysfunction, skin rash, HMB, and HV.^7^ Less frequent symptoms are pancytopenia, central nervous system involvement, digestive disorders, parotitis, and oral ulcer. Additionally, CAEBV could result in life-threatening complications, such as hemophagocytic syndrome, malignant lymphoma, hepatic failure, digestive tract perforation, myocarditis, and interstitial pneumonia.^7^ Typical cases are not difficult to be recognized, however, patients with atypical manifestations are easily misdiagnosed.

In this article, we present an uncommon juvenile case of EBV-positive T/NK-cell LPD manifested as gastrointestinal disorders and skin lesions, who experienced repeatedly spontaneous intestinal perforations. The patient was originally misdiagnosed as inflammatory bowel diseases (IBD) and treated accordingly. He was not diagnosed as CAEBV correctly until the disease progressed to lymphoma stage. This is a rare case that could be a good lesson for clinicians.

## CASE REPORT

**FIGURE 1:**
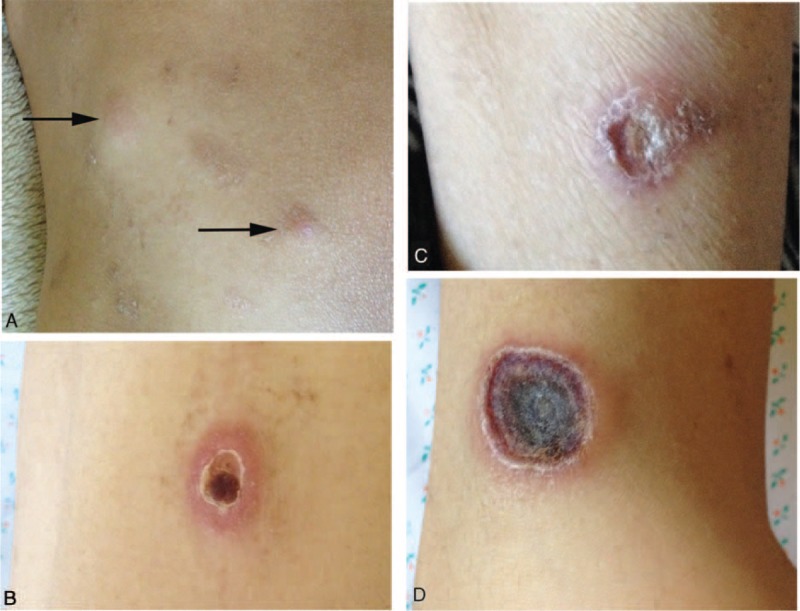
The skin lesions of the patient. Cutaneous nodules in the buttock (A). Skin lesions in the upper right arm (B), lower right leg (C), and lower left leg (D).

A 14-year-old boy was admitted for the first time to our hospital, complaining of a chronic bellyache and diarrhea, intermittent fever, and occasional hematochezia lasting 1 year and 9 months. He experienced severe right lower abdominal pains, was diagnosed “appendicitis,” and received “appendicectomy” 4 months before hospitalization. In the last 2 months, he suffered from more frequent fever and hematochezia, obvious weight loss (from 40 to 30 kg), and skin nodules in four limbs and buttock, which ruptured and scabbed gradually (Figure 1). He was diagnosed as IBD in local hospital, receiving mesalazine and prednisone (10 mg/day) for 1 month. He had no history of other diseases and no related family history. On physical examination, his lean appearance, superficial lymphadenopathy, and hepatosplenomegaly were noted.

Laboratory tests showed severe microcytic hypochromic anemia and hypoalbuminemia, with the hemoglobin (HGB) and serum albumin of 58 g/L (110–150) and 28 g/L (35–52), respectively. The patient suffered from decreased serum total calcium of 1.93 mmol/L (2.13–2.70) and 25-hydroxy vitamin D of 6.8 ng/ml (8–50), and elevated erythrocyte sedimentation rate (ESR) of 38 mm/hour (0–15) and C-reactive protein (CRP) of 40 mg/L (0–8). His serum ferrium, total iron binding capacity, transferrin saturation, and ferritin were all decreased. Fecal occult blood was positive, and no red and white blood cells were found in the stool. Salmonella typhi was positive from stool culture; Widal reaction was negative. T-SPOT.TB test (an assay used for tuberculosis diagnosis) was weakly positive; procalcitonin (PCT), G-test (a detection method for fungal infections), and antibodies to EBV and cytomegalovius were all negative. Autoantibodies related to IBD and autoimmune diseases were all negative. There were no evidence for tumor from bone marrow smear, tumor markers, and whole body diffusion-weighted imaging (DWI).

The abdominopelvic enhanced computed tomography (CT) scanning demonstrated bowel wall thickening, abnormally strengthening mucosal signals, and coarse serous membranes of terminal ileum, caecum, and ascending colon. Multiple enlarged abdominal lymph nodes, small intraabdominal effusions, and calcifications inside the liver and scrotum were also demonstrated. The previous colonoscopy showed ulcers and erosions; pathology showed chronic mucositis accompanied with exudation and necrosis. In order to identify his primary diagnosis, we performed skin biopsy and prepared to perform the second colonoscopy. Meanwhile, we continued his previous treatment and gave his symptomatic and supportive treatment.

**FIGURE 2:**
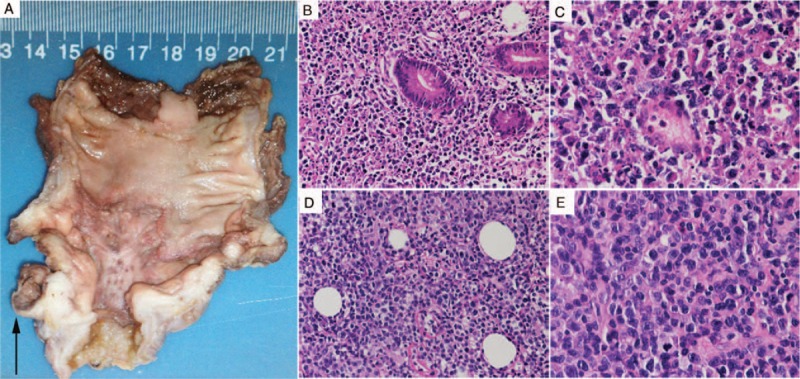
Pathological results of intestine and skin biopsy. The perforation in ileocecus (A). HE staining showed diffuse heterotypic lymphoid cells infiltration, karyorrhexis, and patchy necrosis in the intestine (B, ×200; C, ×400) and skin (D, ×200; E, ×400). HE = hematoxylin–eosin.

However, he suddenly suffered from perforation in ileocecus (severe abdominal pain, tachycardia, and subdiaphragmatic free air). The emergency resection and anastomosis was successful, and the resected ileocecus was sent for pathology (Figure 2A). Several antibiotics and related treatment were added. However, on the third day after surgery, he experienced severe abdominal pain again, and excrement was found in his abdominal cavity drainage tube. Surgeons considered the second spontaneous perforation and performed the second operation. However, no gastrointestinal perforation was found, and it is incredible that there were still intestinal substances outflowing from the abdominal cavity drainage tube after surgery. His parents refused surgical treatment further; we continued medical conservative treatment.

**FIGURE 3:**
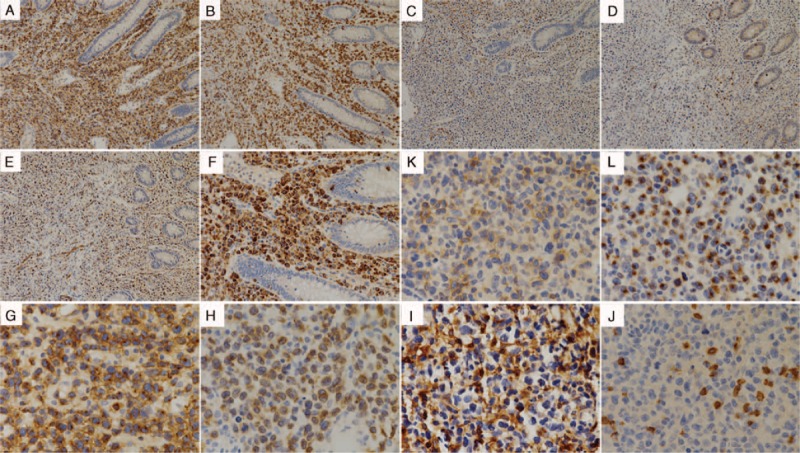
Immunohistochemical staining results of the intestine (A–E, ×100; F, ×200) and skin (G–L, ×400) biopsy. (A and G) CD2+. (B and H) CD3+. (C and I) CD4−. (D and J) CD8−. (E and K) CD56±. (F and L) GrB+. CD = cluster of differentiation; GrB = granzyme B.

**FIGURE 4:**
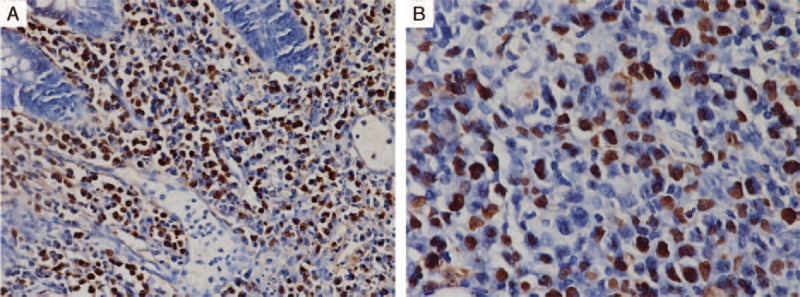
Positive FISH for EBER in the intestine (A, ×200) and skin (B, ×400). EBER = EBV-encoded early small RNA; EBV = Epstein–Barr virus; FISH = fluorescence in situ hybridization; RNA = ribonucleic acid.

The boy died from massive intestinal hemorrhea after a week. The pathological results of intestine and skin both showed diffuse heterotypic lymphoid cells infiltration; karyorrhexis and patchy necrosis could also be seen (Figure 2B–E). Combined with immunohistochemical staining (cluster of differentiation 2 (CD2)+, CD3+, CD5−, CD20−, CD4−, CD8−, CD30+ most, CD56±, T-cell restricted intracellular antigen 1 (TIA-1)+, granzyme B (GrB)+, T-cell receptor αβ (TCRαβ)−, TCRγδ−, and KI-67+ >50%; Figure 3) and positive fluorescence in situ hybridization (FISH) for EBV-encoded early small ribonucleic acid (RNA) (EBER, Figure 4) both in the intestine and skin, EBV-positive T/NK-cell LPD (tumor stage) was identified.

## DISCUSSION

According to the patient's clinical features and laboratory indicators, his primary diagnosis was not clear; several possibilities were considered. IBD came to our mind first, which could explain all his manifestations. However, there were no typical IBD manifestations from his first colonoscopy and pathological results, such as longitudinal ulceration, cobble-stoning, crypt architecture distortion, and so on.^8^ What is more, his skin lesions were not the typical cutaneous manifestations of IBD. He suffered from obvious body weight loss in a short time, and his skin lesions manifested as nodules, ruptures, and escharosis. Tumor, especially lymphoma that could explain all of his clinical features, should also be considered. However, he experienced such a long illness course that it is very difficult to explain it only by lymphoma. Meanwhile, his previous colonoscopy biopsy pathology only showed mucositis (no malignant manifestations).

His positive T-SPOT.TB test and stool culture made us think of enterophthisis and typhoid fever, which could not explain skin lesions though. False positive rate of T-SPOT.TB test is 7%, and this patient's test was only weakly positive.^9^ What is more, he had no tuberculosis infection of other locations, and his colonoscopy pathology did not show caseous necrotizing granuloma. Widal test is of more limited clinical utility compared with stool culture, one of the laboratory indicators on which diagnosis of typhoid fever is largely dependent.^10^ However, he had no contact history of typhoid, and his disease course was so long that typhoid fever as the primary disease could not explain it. We should repeat related examinations and give preventive treatment if needed. As for this patient, the primary disease complicated with salmonella typhi infection could not be excluded. We gave him appropriate antibiotics.

The boy was eventually diagnosed as EBV-positive T/NK-cell LPD (lymphoma stage). There have been some reports about gastrointestinal perforations in non-Hodgkin's lymphomas (NHL), which are more frequently associated with aggressive than indolent lymphomas.^11^ Several studies reported an inferior outcome of lymphomas when complicated by perforation.^12^ However, there are rare studies reporting gastrointestinal perforations in EBV-positive T/NK-cell LPD, let alone repeatedly spontaneous perforations. It is quite strange that surgeons did not find any perforations in the second operation. Maybe, the lesions of his gastrointestinal tract were so severe that there were diffuse small perforations in small intestines or even colon, which could not been recognized by eyes.

According to the 2008 WHO lymphoma classification, this patient was diagnosed as systemic EBV-positive T-cell LPD of childhood. However, how to explain the whole illness courses based on its highly aggressive feature? How to explain the previous colonoscopy biopsy pathological result (mucositis)? CAEBV should be considered for the close association with systemic EBV-positive T-cell LPD of childhood.^5^ For our patient, CAEBV could be diagnosed by a more than 1.5-year disease course, recurrent fevers, hepatosplenomegaly and lymphadenopathy in hospitalization, digestive tract and dermal complications, and positive EBER in intestine and skin.^13^ We have reason to believe that the patient's disease course developed from CAEBV (T-cell type) to systemic EBV-positive T-cell LPD of childhood (lymphoma), which is consistent to the conclusion in 1 Japanese literature that EBV-positive T/NK-cell LPDs in children progress from a polyclonal, to oligoclonal, to monoclonal EBV-driven proliferation.^3^

Patients with CAEBV generally have high anti-viral capsid antigen (VCA) and anti-early antigen (EA) titers. Additionally, most patients are anti-EB nuclear antigen (EBNA) antibody positive.^7^ However, it should be noted that not all patients present unusual patterns of anti-EBV antibodies and that high antibody titers against these EBV-related proteins are not necessary for the diagnosis of CAEBV; the presence of high EBV loads in affected tissues and/or peripheral blood is essential for diagnosing CAEBV.^13,14^ It is reported that elevated immunoglobulin G (IgG) antibodies against EBV-lytic antigens such as VCA and EA indicate increased EBV replication, and that an EBV-immortalized cell expresses viral proteins including the highly immunogenic EBNA which are major targets of EBV-specific cytotoxic T lymphocytes (CTL).^6^ Lack of expression of these antigens in CAEBV individuals might suggest the presence of both abnormal cellular regulation and defective of unbalanced immunosurveillance.^6^ For our patient, his negative EBV-related antibodies might be related to the false negative detection result or his own individual factors.

The initial and distinct manifestations of this patient were gastrointestinal disorders and recurrent fevers, when he was treated as gastroenteritis or IBD all the time and could remain a stable condition for more than 1.5 years. Maybe based on his less common clinical features for CAEBV and negative EBV-related antibodies, his EBV DNA in peripheral blood was not tested, FISH for EBER was not tested in the intestinal biopsy (by colonoscopy) pathological examination either, and no doctors considered EBV infection. We should have emphasized the tests for EBV DNA in peripheral blood and EBER-positive cells in affected tissues.

The effect of the treatment (mesalazine and prednisone of suitable doses) was unsatisfactory in our patient, who still suffered from severe digestive and systemic disorders. In fact, antiviral treatment, immunoenhancers such as interferons, immunosuppressants, and antiproliferative chemotherapies all have limited effects for CAEBV.^6^ To date, only hematopoietic stem cell transplantation (HSCT) has been shown to be promising for EBV-positive T/NK-cell LPD patients, including those not yet having progressed to lymphoma.^6^ For the patients diagnosed as IBD, who did not have the typical skin, colonoscopy, and pathological manifestations, and whose clinical symptoms were not obviously improved after receiving the treatment against IBD, we should doubt the diagnosis, consider the possibility of infectious or malignant diseases, and carry out further related examinations.

In summary, we described an uncommon young case of EBV-positive T/NK-cell LPD who was misdiagnosed as IBD for a relatively long time for his prominent digestive disorders. The less common manifestations for CAEBV and undetectable EBV-related antibodies made the correct diagnosis very difficult. We should realize the importance of the tests for EBV DNA in peripheral blood and EBER in affected tissues. Meanwhile, for the IBD patients whose manifestations were not typical, and whose conditions were not improved by suitable therapies against IBD, infectious and malignant diseases should be considered.

Informed consent for publication of this case was obtained.
