# Epstein–Barr Virus-Positive T/NK-Cell Lymphoproliferative Disorders Manifested as Gastrointestinal Perforations and Skin Lesions: A Case Report: Erratum

**DOI:** 10.1097/01.md.0000482728.66173.42

**Published:** 2016-04-18

In the article “Epstein–Barr Virus-Positive T/NK-Cell Lymphoproliferative Disorders Manifested as Gastrointestinal Perforations and Skin Lesions: A Case Report”,^1^ which appeared in Volume 95, Issue 5 of *Medicine*, the locants B and C in Figure 1 were mismatched. The article has since been corrected online.
